# A Chromosome-level Reference Genome of African Oil Palm Provides Insights into Its Divergence and Stress Adaptation

**DOI:** 10.1016/j.gpb.2022.11.002

**Published:** 2022-11-24

**Authors:** Le Wang, May Lee, Zi Yi Wan, Bin Bai, Baoqing Ye, Yuzer Alfiko, Rahmadsyah Rahmadsyah, Sigit Purwantomo, Zhuojun Song, Antonius Suwanto, Gen Hua Yue

**Affiliations:** 1 Temasek Life Sciences Laboratory, Singapore 117604, Singapore; 2 Wheat Research Institute, Gansu Academy of Agricultural Sciences, Lanzhou 730070, China; 3 Biotech Lab, Wilmar International, Bekasi 17530, Indonesia; 4 R & D Department, Wilmar International Plantation, Palembang 30118, Indonesia; 5 Department of Biological Sciences, National University of Singapore, Singapore 117558, Singapore

**Keywords:** Oil palm, Genome, Evolution, *VIRESCENS*, Molecular breeding

## Abstract

The palm family (Arecaceae), consisting of ∼ 2600 species, is the third most economically important family of plants. The African **oil palm** (*Elaeis guineensis*) is one of the most important palms. However, the **genome** sequences of palms that are currently available are still limited and fragmented. Here, we report a high-quality chromosome-level reference genome of an oil palm, *Dura*, assembled by integrating long reads with ∼ 150× genome coverage. The assembled genome was 1.7 Gb in size, covering 94.5% of the estimated genome, of which 91.6% was assigned into 16 pseudochromosomes and 73.7% was repetitive sequences. Relying on the conserved synteny with oil palm, the existing draft genome sequences of both date palm and coconut were further assembled into chromosomal level. Transposon burst, particularly long terminal repeat retrotransposons, following the last whole-genome duplication, likely explains the genome size variation across palms. Sequence analysis of the ***VIRESCENS*** gene in palms suggests that DNA variations in this gene are related to fruit colors. Recent duplications of highly tandemly repeated pathogenesis-related proteins from the same tandem arrays play an important role in defense responses to *Ganoderma*. Whole-genome resequencing of both ancestral African and introduced oil palms in Southeast Asia reveals that genes under putative selection are notably associated with stress responses, suggesting adaptation to stresses in the new habitat. The genomic resources and insights gained in this study could be exploited for accelerating genetic improvement and understanding the **evolution** of palms.

## Introduction

The palm family (Arecaceae) consists of ∼ 2600 species belonging to over 180 genera [1]. Over 90% of the diversity within this family is distributed in the tropical region of the world by adaptive radiation [2]. The Arecaceae is the third most economically important family of plants, after grasses and legumes [3]. The African oil palm (*Elaeis guineensis*) is the most economically important of the Arecaceae, with a global production of ∼ 74 million metric tons of vegetable oil (FAOSTAT, https://www.fao.org/faostat, accessed at 2021/05/03). African oil palm is native to West Africa from Angola northward to Gambia [4]. It was introduced to Southeast Asia in the 1840s, and has been naturalized since then [4]. Oil palm is the most productive oil plant and produces over 35% of vegetable oils with a market value of over $40 billion (EPOA, https://www.palmoilandfood.eu/en/palm-oil-production, accessed at 2022/05/10). Although the oil yield has improved from ∼ 2.0 tons/ha/yr to the current ∼ 4.0 tons/ha/yr in the past 100 years, there is still great potential to further improve the oil yield and other economical traits [4]. In addition, the oil palm industry is seriously threatened by diseases caused by the *Ganoderma* species, resulting in losses of up to 80% of yield in some plantation areas [4]. Improvement of economically important traits using various approaches, including conventional and molecular breeding, is critically important in the oil palm industry.

A high-quality genome assembly is necessary for both molecular breeding to accelerate genetic improvement and understanding species’ evolution. Despite the need to better understand oil palm genomics, only draft genome sequences are available. The completeness and quality of the published genome assemblies are still to be improved [5], [6], [7]. Only ∼ 60% of the 1.8-Gb estimated genome sequences were assembled, and ∼ 45% of sequences were anchored to genetic maps in *Pisifera* genome version EG5.1 and/or PMv6 [6], [7]. These draft genome sequences supply important resourses to initiate molecular breeding to accelerate the genetic improvement. However, due to the limited completeness, fragmentation of scaffolds, and incomplete annotations, their applications in genome-wide association studies, comparative genomics, and structural variation analysis in the oil palm species and their related species are limited. Therefore, further improvement of the draft genome of oil palm is essential for molecular breeding in order to improve economic traits and understand the evolution of palms through comparative genomics [8].

Here, we report a high-quality chromosome-level genome assembly of *E. guineensis*. Comparative genomic analysis revealed that transposon burst was responsible for genome size expansion in palms. We found evidence that highly tandemly repeated pathogenesis-related (PR) proteins played an important role in defense responses to *Ganoderma* infection. Whole-genome resequencing of 72 trees from West Africa and Southeast Asia revealed the population structure and lower genetic variations of oil palms in Southeast Asia. Signatures of local adaptation in the genome of oil palm was also found. The novel genomic resources and insights gained from this study will contribute to the understanding of palm evolution and accelerate the genetic improvement of oil palm.

## Results and discussion

### Chromosomal-level genome of African oil palm

**Figure 1:**
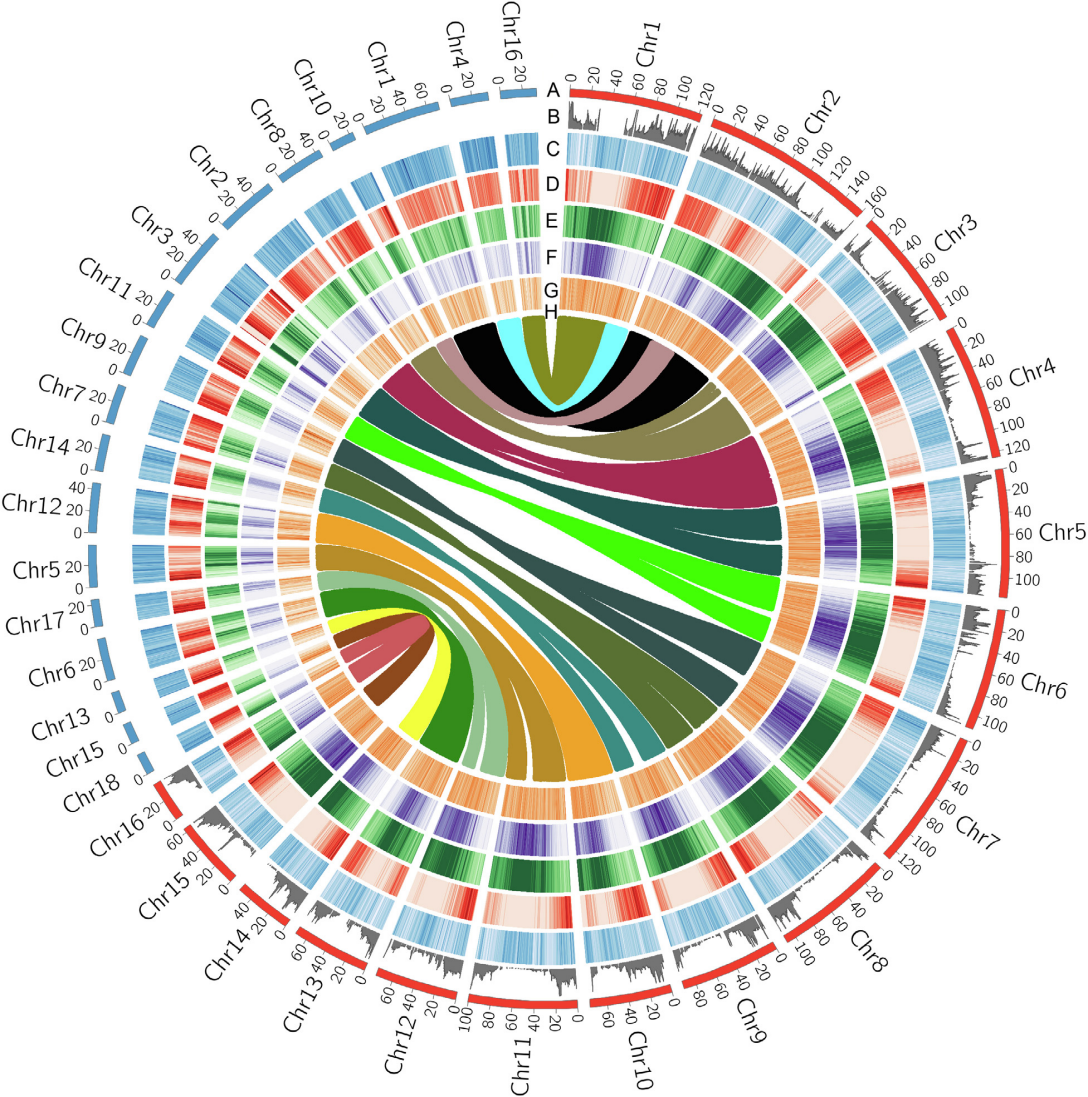
**Global view of genomic features of oil palm and genomic synteny with date palm** **A.** Length of individual pseudochromosomes (Mb). **B.** Distribution pattern of recombination rate throughout individual chromosomes. **C.** Distribution pattern of GC content throughout individual chromosomes, estimated in 500-kb window. **D.** Distribution pattern of gene density throughout individual chromosomes, estimated in 500-kb window. **E.** Distribution pattern of repetitive sequences throughout individual chromosomes, estimated in 500-kb window. **F.** Distribution pattern of LTR retrotransposon superfamily *Copia* throughout individual chromosomes, estimated in 500-kb window. **G.** Distribution pattern of LTR retrotransposon superfamily *Gypsy* throughout individual chromosomes, estimated in 500-kb window. **H.** Conserved syntenic blocks between a pair of homologous chromosomes of oil palm and date palm. Chr, chromosome; LTR, long terminal repeat.

**Supplementary Figure S1:**
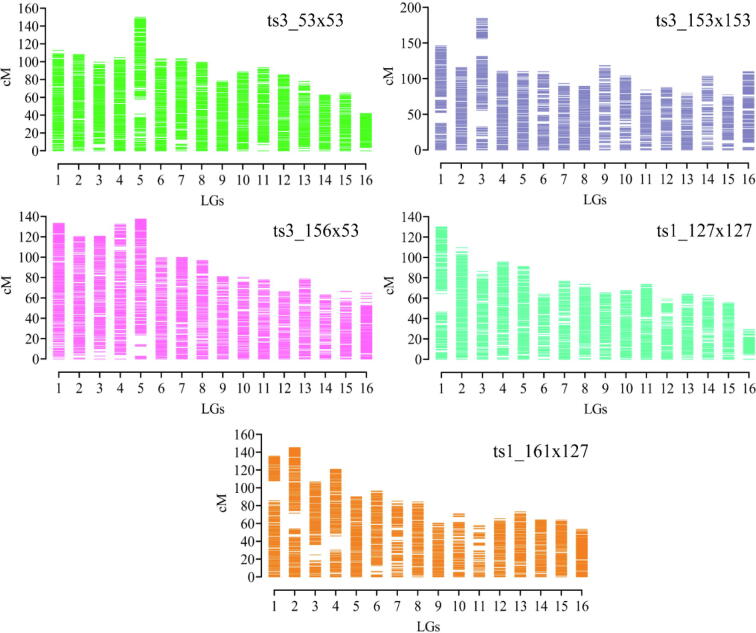
Distribution of SNPs in the five high-density linkage maps of the African oil palm SNPs, single nucleotide polymorphisms; LGs, linkage groups

**Supplementary Figure S2:**
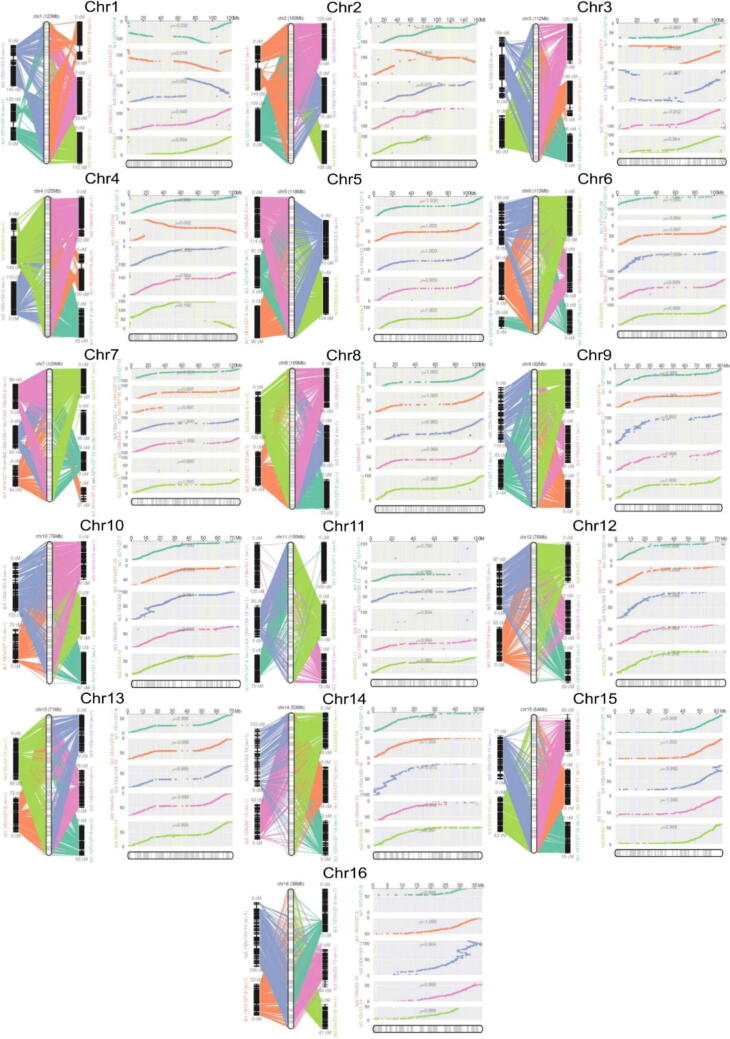
Anchoring contigs to five high-density linkage maps of the African oil palm, using the program ALLMAPS Pearson’s correlation coefficient between each linkage group and the corresponding physical maps is denoted with ρ value

Over 150× coverage of long reads was assembled into 4752 contigs, with a total length of 1.7 Gb, covering 94.5% of the estimated genome (1.8 Gb) (Table S1). Contig N50 and the longest contig reached up to 2.168 Mb and 12.851 Mb, respectively. We constructed five high-density linkage maps in five F_2_ populations, with the number of mapped markers ranging from 12,068 to 19,581 (Figure S1; Table S2). Anchoring contigs to these high-density genetic maps, based on a total number of 60,989 informative segregating markers, resulted in 16 pseudochromosomes consisting of 91.6% of assembled sequences and with length ranging from 37.784 Mb to 160.148 Mb, and 59.7% of assembled sequences were oriented (Figure S2; Tables S1, S3, and S4). Genome completeness analysis assessed with Benchmarking Universal Single-Copy Orthologs (BUSCO) showed that 95.8% of the core genes were found in the genome and 93.3% were complete (Table S5). Mapping of assembled transcripts and *de novo* assembled restriction-site associated DNA (RAD) tags showed that 98.8% and 97.5% were matched to the genome assembly, respectively. We annotated long terminal repeats (LTRs). The LTR assembly index (LAI) was estimated to be 15.453 ± 2.968 (mean ± standard deviation). This genome assembly significantly increases the total length of assembled sequences by ∼ 61% (contig length from ∼ 1057 Mb to ∼ 1701 Mb), N50 contig size of ∼ 233 folds, N50 scaffold size of ∼ 80 folds, and total length of sequences anchored on pseudochromosomes of ∼ 2.4 folds, compared with previous draft genome sequences (Table S1) [5], [6], [7]. A chromosomal-level genome is necessary for comparative genomic analysis to study genome duplications and understand the genomic architecture of adaptive radiation of palms. Date palm (*Phoenix dactylifera*) Barhee BC4 is one of the most impressive assemblies in palms, in which < 50% of sequences were anchored to pseudochromosomes [9]. Although diverged ∼ 65 million years ago (MYA) [6], we observed a high level of conserved chromosome synteny between oil palm and date palm (Figure 1), indicating that the chromosomal-level genome of oil palm can be used for comparative genomic analysis. Taken together, our genome assembly showed high genome coverage, high assembly accuracy, long sequence continuity, and high completeness of both genes and repetitive elements. Therefore, it will be a vital contribution to studies on genetics, genomics, and breeding in palm species.

### Annotation of the African oil palm genome

Repetitive sequences accounted for ∼ 74% of the genome assembly of oil palm (Table S6), significantly higher than that previously observed in the incomplete genome assembly of this species (∼ 57%) [6] and that in date palm (∼ 39%) [9]. LTRs took up 55.79% of the genome. *Copia* is the largest class of LTRs, followed by the *Gypsy* superfamily, representing 39.46% and 17.19% of the assembled genome sequences, respectively (Table S7). The proportions of the two LTR superfamilies are also much higher than those in date palm (∼ 14% for *Copia* and ∼ 4% for *Gypsy*) [9]. We observed that the distribution pattern of repetitive sequences was negatively correlated to that of the recombination rate (*R* = −0.412, *P* < 1 × 10^−4^) and the gene density (*R* = −0.794, *P* < 1 × 10^−6^), but positively correlated to the distribution pattern of GC content (*R* = 0.932, *P* < 1 × 10^−6^) (Figure 1A–E). In date palm, we observed the same correlation patterns between the repetitive sequences and gene density (*R* = −0.856, *P* < 1 × 10^−6^) and between the repetitive sequences and GC content (*R* = 0.403, *P* < 1 × 10^−6^) (Figure 1A–E) as in oil palm, which addresses how transposon dynamics has significantly shaped the genomic architecture of palms. We observed that the distribution of *Copia* was highly correlated with that of the overall repetitive sequences (*R* = 0.952, *P* < 1 × 10^−6^), whereas *Gypsy* were more likely randomly distributed across the genome (*R* = 0.107, *P* < 0.05) (Figure 1F and G). Our data indicate that palms have a much higher copy number of *Copia* compared with *Gypsy*, contradicting most other plant genomes, which show higher *Gypsy* content [9]. Previous studies have reported that retrotransposons in plants play important roles in genome size, genome structure remodeling, gene function, and genome evolution [10]. Therefore, it is highly possible that *Copia* may play an important role in the evolution of palms.

**Supplementary Figure S3:**
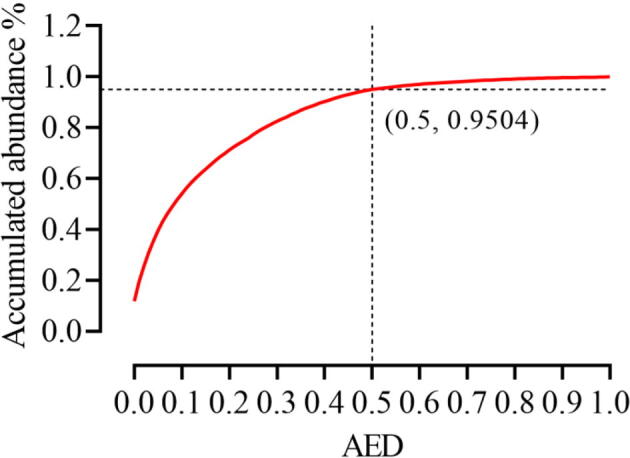
Distribution of the AED values throughout all predicted protein coding genes of the African oil palm genome AED, annotation edit distance

**Supplementary Figure S4:**
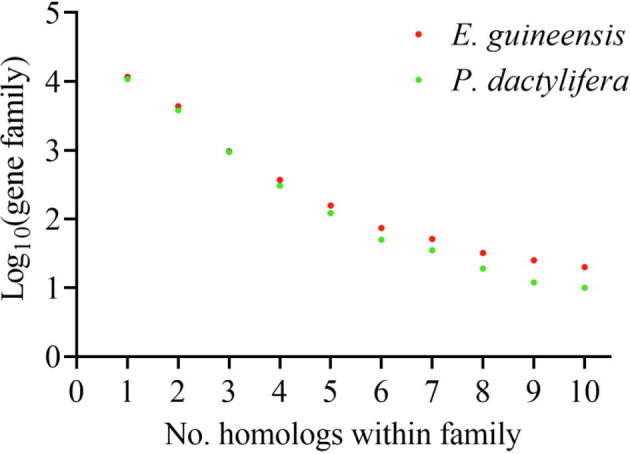
Comparison of expanded gene families between the African oil palm and date palm

**Supplementary Figure S5:**
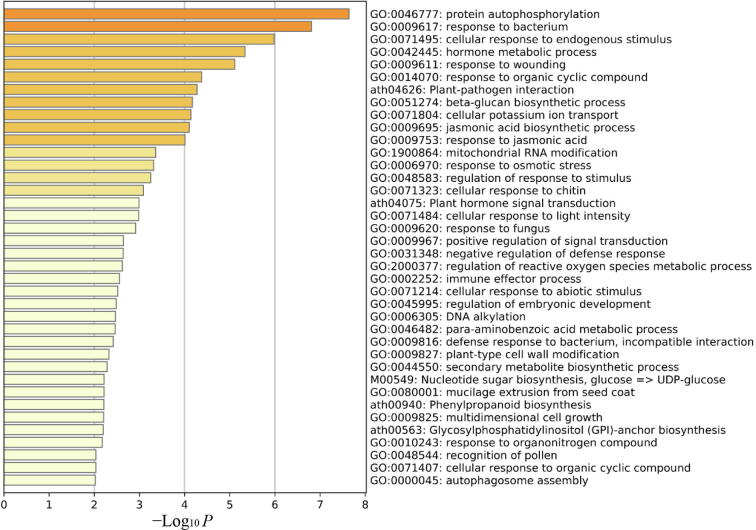
Functional enrichment of the expanded gene families in the African oil palm, compared to date palm

Gene annotations based on RNA sequencing (RNA-seq), *ab initio* predictions, plant protein-coding genes, and protein domains, predicted 33,447 protein-coding genes. Of these genes, 29,293 (87.58%) were annotated with known proteins or domains (Table S1). Over 95% of predicted genes showed an annotation edit distance (AED) value of < 0.5, indicating high-quality annotations of the genome (Figure S3). Median gene length was ∼ 5.2 kb, slightly higher than those of previous oil palm and date palm assemblies of ∼ 4.7 kb and ∼ 4.2 kb, respectively [6], [9]. In addition, more than 98% of the annotated genes were mapped to the 16 chromosome sequences, indicating that this genome assembly represents a nearly complete protein-coding genome and is useful in future genetic and genomic studies. Functional enrichment analysis revealed that gene families showing expansions in oil palm were more involved in stress responses to pathogens and regulation of osmotic stresses (Figures S4 and S5).

### Transposon burst leads to genome expansion and gene diversification in palms

**Figure 2:**
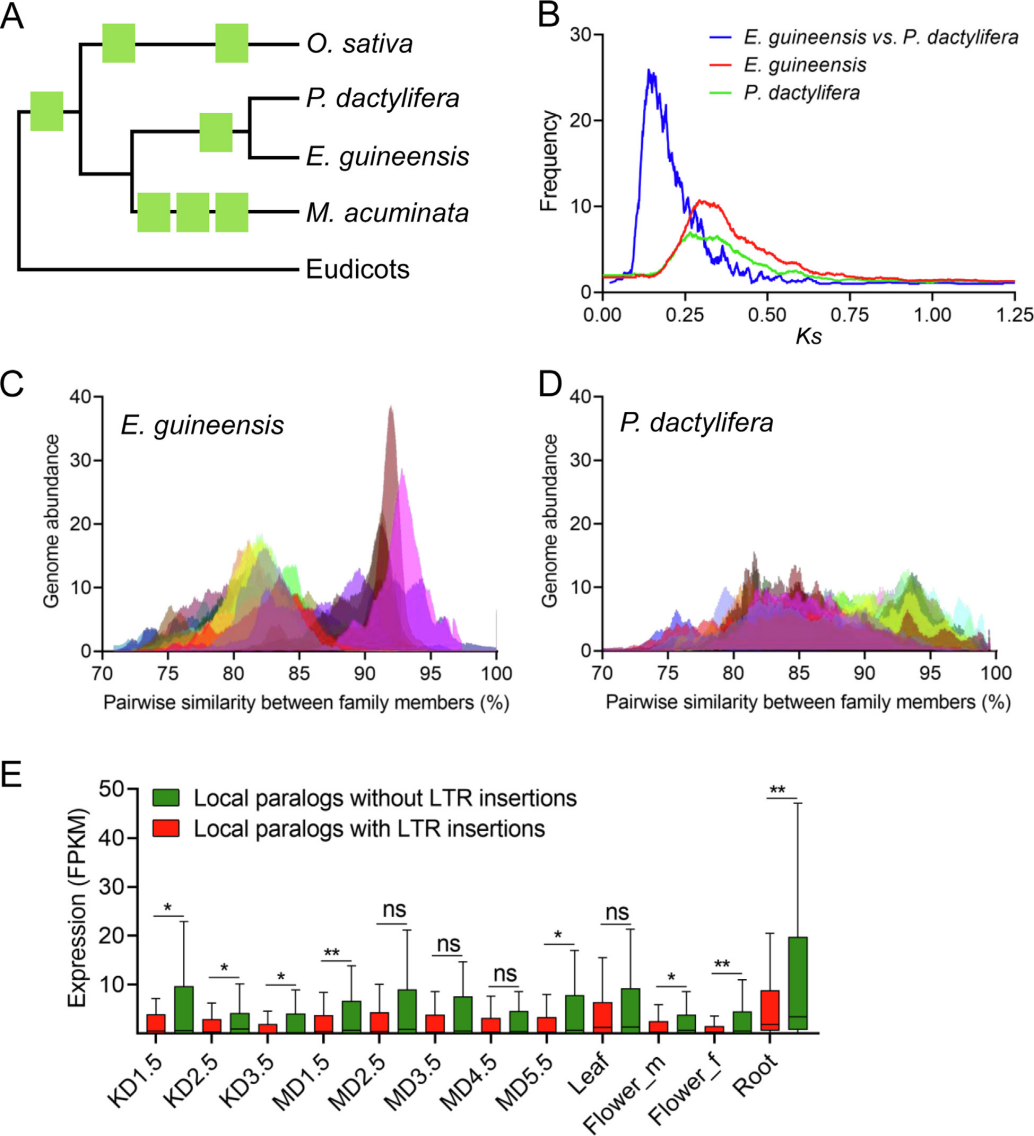
**Transposon expansion drives genome evolution of palms** **A.** A phylogram showing historical WGD events in palms. **B.** Distribution of *Ks* between a pair of homologous genes between oil palm and date palm (speciation) and separately within oil palm and date palm (WGD). **C.** Pairwise transposon divergence throughout 30 randomly selected subfamilies of LTR retrotransposon superfamily *Copia* in oil palm. Two major peaks at an average similarity of ∼ 82% and ∼ 92% are revealed. **D.** Pairwise transposon divergence throughout 30 randomly selected subfamilies of LTR retrotransposon superfamily *Copia* in date palm. Two major peaks at an average similarity of ∼ 84% and ∼ 93% are only slightly visible. **E.** Comparison of the relative expression levels between a pair of locally duplicated genes throughout 12 examined samples. Only one of each pair of paralogous genes shows intact LTR insertion in gene feature. KD1.5, KD2.5, and KD3.5 indicate kernel samples at 1.5, 2.5, and 3.5 months after fertilization, respectively, while MD1.5, MD2.5, MD3.5, MD4.5, and MD5.5 indicate mesocarp samples at 1.5, 2.5, 3.5, 4.5, and 5.5 months after fertilization, respectively. Flower_m and Flowe_f represent male and female flowers, respectively. *, *P* < 0.05; **, *P* < 0.01; ns, not significant (paired *t*-test). WGD, whole-genome duplication; *Ks*, synonymous substitution rate; FPKM, fragments per kilobase of transcript per million mapped reads; *E. guineensis*, *Elaeis guineensis*; *P. dactylifera*, *Phoenix dactylifera*; *O. sativa*, *Oryza sativa*; *M. acuminata*, *Musa acuminata.*

**Supplementary Figure S6:**
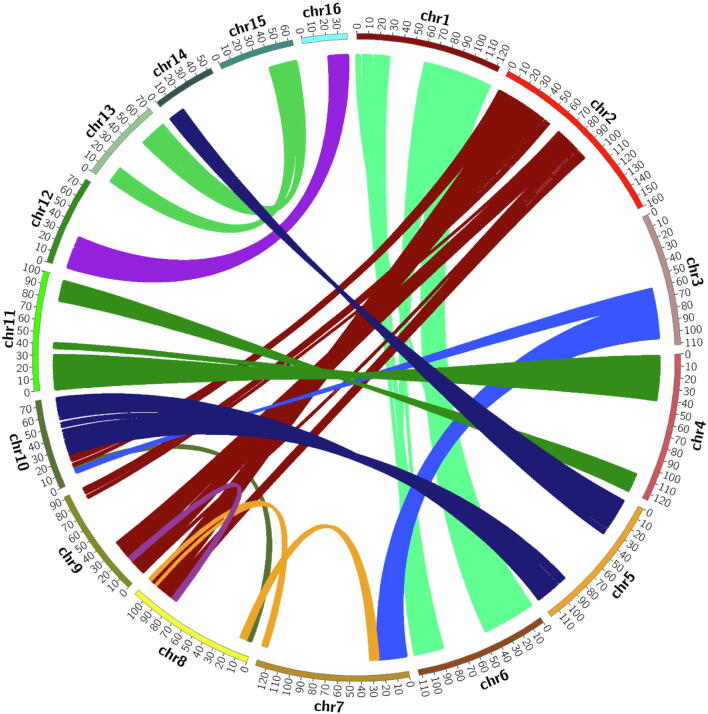
Circos plot of homologous blocks between a pair of chromosome fragments in the African oil palm

The variation in genome size across eukaryotes is tremendous and is associated with species diversity [11]. Polyploidy and transposon expansion are the two major forces driving genome size variation, providing essential resources for evolutionary innovations by generating novel genetic variations and altering gene expression patterns [12]. Thus, it is necessary to unravel these mechanisms in order to better understand adaptive radiation and successful ecological dominance of the taxa. Genome size of palms varies from ∼ 800 Mb to ∼ 3 Gb [13]. Oil palm and date palm show a striking difference in genome size, with the predicted size of 1.8 Gb and 800 Mb, respectively, providing an excellent system to study genome size variation. Monocots share a common whole-genome duplication (WGD) event at ∼ 150 MYA [14]. The other paleopolyploid event, exclusively for the ancestor of all palms, occurred at ∼ 75 MYA, resulting in the paleotetraploidy of all palms [6], [13]. We observed large conserved syntenic blocks between homologous chromosome pairs throughout the whole genome (Figure S6), allowing for examination of the effects of WGD events on genome evolution. Distribution of synonymous substitution rate (*Ks*), estimated based on 4292 and 2793 pairs of homologous genes from syntenic blocks in oil palm and date palm, respectively, revealed a major peak at ∼ 0.32, corresponding to the recent WGD at ∼ 75 MYA that was shared by all palms (Figure 2A and B) [13]. A more recent *Ks* peak was observed at ∼ 0.22 for orthologous gene pairs, indicating the divergence between oil palm and date palm at ∼ 65 MYA [6]. Here, the divergence of the whole-genome-wide homologous genes supports the conclusion that all palms have experienced two WGD events before adaptive radiation [13].

**Supplementary Figure S7:**
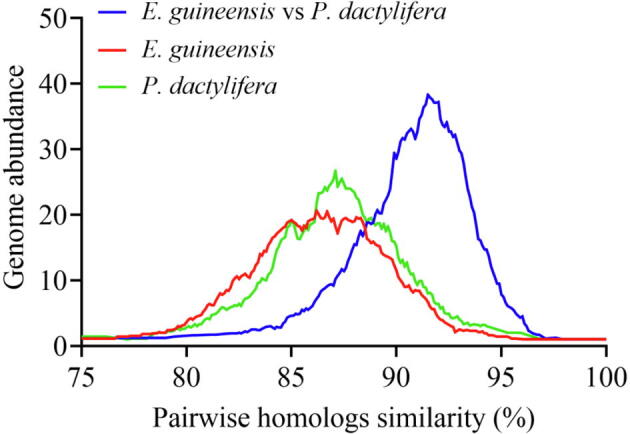
Distribution of pairwise sequence divergence between homologous genes Pairwise sequence divergence was estimated within oil palm and date palm (WGD event), and between oil palm and date palm (speciation event)

We did not find notable evidence of gene loss in date palm in contrast to oil palm, leading to another hypothesis that transposon proliferation drives genome size expansion and speciation of palms. LTRs are the richest transposable elements (TEs) in both species, with a total length of ∼ 950 Mb and ∼ 200 Mb [9] for oil palm and date palm, respectively. Difference in LTR content explains ∼ 80.3% of the genome size variation between the two species. Among LTRs, *Copia* is the most abundant superfamily for both species and accounts for ∼ 52.3% of total genome size variation (Table S7). We examined the historical dynamics of each subfamily of *Copia*. Pairwise sequence divergence within each subfamily presented two peaks with sequence similarity of ∼ 82% and ∼ 92%, respectively, in oil palm (Figure 2C). In comparison, two peaks of sequence divergence in date palm at ∼ 84% and ∼ 93% were only slightly visible (Figure 2D). In particular, the peak of higher similarity was remarkably inflated in oil palm, suggesting a recent transposon burst in oil palm relative to date palm. We compared the sequence divergence between *Copia* and homologous genes that mark the last WGD and diversification. Homologs in conserved syntenic blocks within each species presented a consistent peak with a similarity of ∼ 86%, whereas the divergence of homologs between species was ∼ 92%, marking the WGD and diversification events, respectively (Figure S7). The first wave of transposon burst, overlapping with the last WGD event, is suggested to be caused by rediploidization due to elevated genomic stress soon after WGD [15]. Under the assumption of comparable sequence evolutionary rates [15], the second wave of transposon burst coincides with the time of divergence between the two palms, suggesting that transposon burst and differential dynamics play an important role in the diversification of palms. Large-scale transposon proliferation and movement may drive chromosome rearrangements, variation of recombination, and gene diversification, and eventually lead to speciation [16].

**Supplementary Figure S8:**
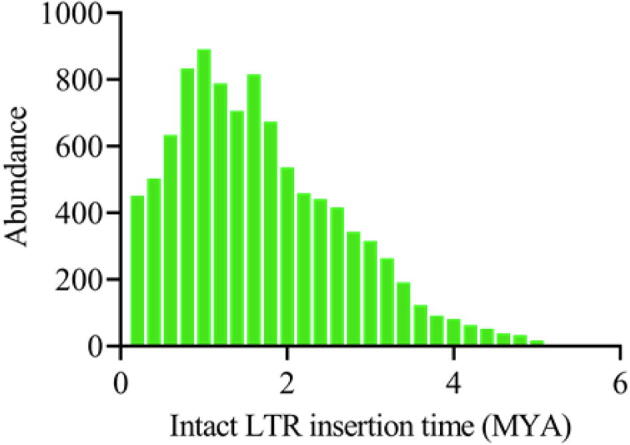
Distribution of estimated LTR insertion time, revealed by LTR_retriever Mya, million years ago

**Supplementary Figure S9:**
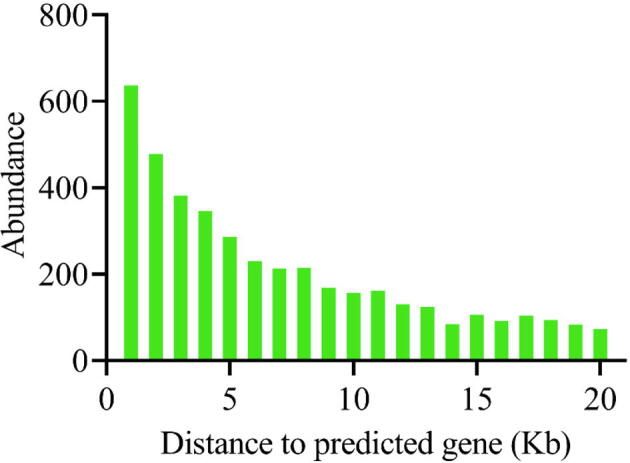
Distribution of the distance between intact LTRs and predicted protein coding genes

**Supplementary Figure S10:**
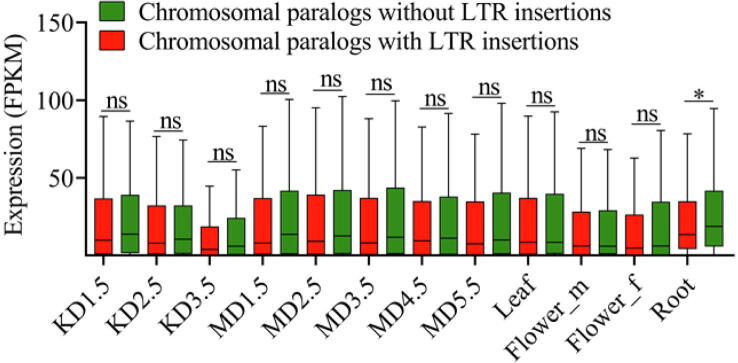
Comparison of the relative expression level between a pair of homologous genes These pairwise homologous genes were from a pair of conserved chromosome syntenic blocks across 12 examined samples, where only one of each pair of homologous genes show intact LTR insertion in the gene feature. KD1.5, KD2.5, and KD3.5 indicate kernel samples at 1.5, 2.5, and 3.5 months after fertilization, respectively, while MD1.5, MD2.5, MD3.5, MD4.5, and MD5.5 indicate mesocarp samples at 1.5, 2.5, 3.5, 4.5, and 5.5 months after fertilization, respectively. * indicates *P* < 0.05 for paired t-test. ns indicates not significant

**Supplementary Figure S11:**
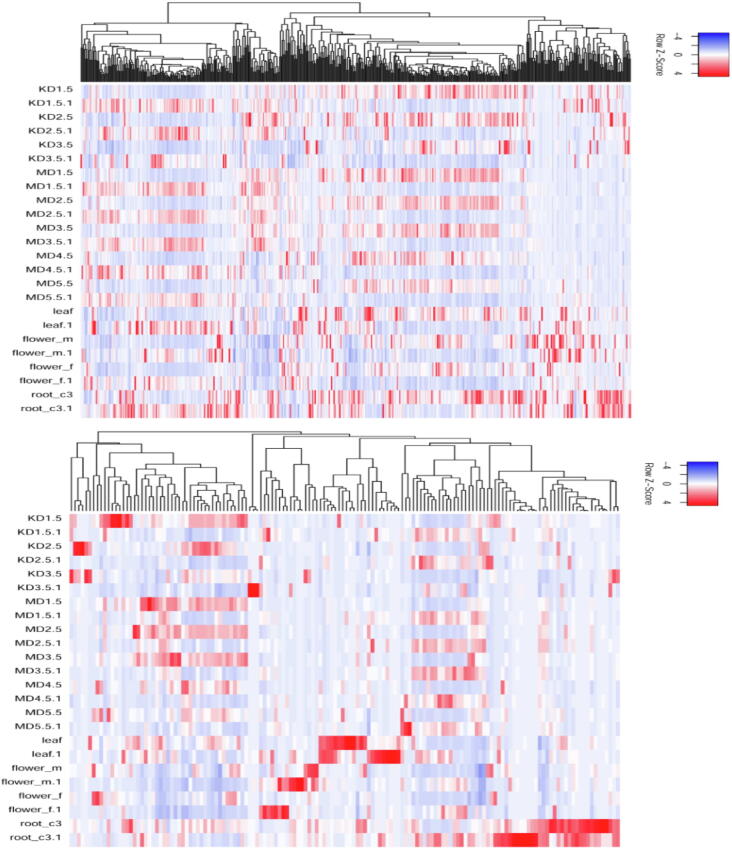
Heatmap shows the relative expression level between a pair of homologous genes These pairwise homologous genes were from a pair of conserved chromosome syntenic blocks across 12 examined samples, where only one of each pair of homologous genes show LTR insertion in the genomic feature (upper figure). The relative expression level between a pair of homologous genes from local duplications across 12 examined samples, where only one of each pair of homologous genes show LTR insertion in the genomic feature (lower figure).

Transposon dynamics can affect genome-wide expression patterns and promote divergence by epigenetic regulation [17]. We identified 9786 intact LTRs throughout the genome. Approximately 33.8% of the intact LTRs showed an estimated insertion time of < 1 MYA (Figure S8), within which 21.8% were in or within 5-kb distance to gene features (Figure S9). We hypothesized that young intact LTRs closely linked to genes can affect gene expression patterns. First, we compared the expression levels of 273 pairs of paralogous genes from conserved syntenic blocks of oil palm, in which only one of a pair of genes is closely linked to a young intact LTR. However, the expression levels of genes linked to LTRs were only slightly reduced (but not significantly) in almost all 12 examined tissues (Figure S10; Table S8). These paralogs diverged since the last WGD at ∼ 75 MYA [14] and likely have functionally diverged in depth. Thus, the effects of LTRs on linked genes are likely underestimated in these anciently duplicated genes. As expected, these paralogous genes presented a more diverse expression pattern than the recent locally duplicated genes (Figure S11). We further examined the effects of intact LTRs on 103 pairs of locally duplicated genes with a younger duplication time (*Ks*, 0.15 ± 0.07). Interestingly, genes linked to LTRs showed significantly lower expression levels as compared with their adjacent paralogs, in most of the examined samples (Figure 2E). Similar results were reported in the tea plant (*Camellia sinensis* var. *sinensis*) [18]. These findings highlight the importance of LTRs in promoting transcriptional diversification of duplicated genes by epigenetic suppression of closely linked genes, which finally contributes to genome divergence.

### Sequence variations in the ***VIRESCENS*** gene may be related to fruit colors in palms

For decades, it has been hypothesized that fruit color had evolved to increase visual conspicuousness, and is subjected to selection by seed dispersing animals [19]. In tropical palms, fruit color evolution is suggested to have interactions with frugivores [20]. However, the genomic basis of adaptive evolution of fruit color in palms is still unclear. *VIRESCENS* encodes a R2R3-MYB transcription factor, which controls the accumulation of anthocyanins in fruit exocarp of palms, leading to deep violet to black fruit colors [21]. The dark pigments of the exocarp reduce the visual conspicuousness in contrast with red, orange, and yellow pigmented fruits, which are caused by carotenoids and carotenes, making it less attractive to herbivorous animals. Thus, genes controlling the accumulation of anthocyanins in the exocarp tend to be under selection by seed dispersing animals.

**Figure 3:**
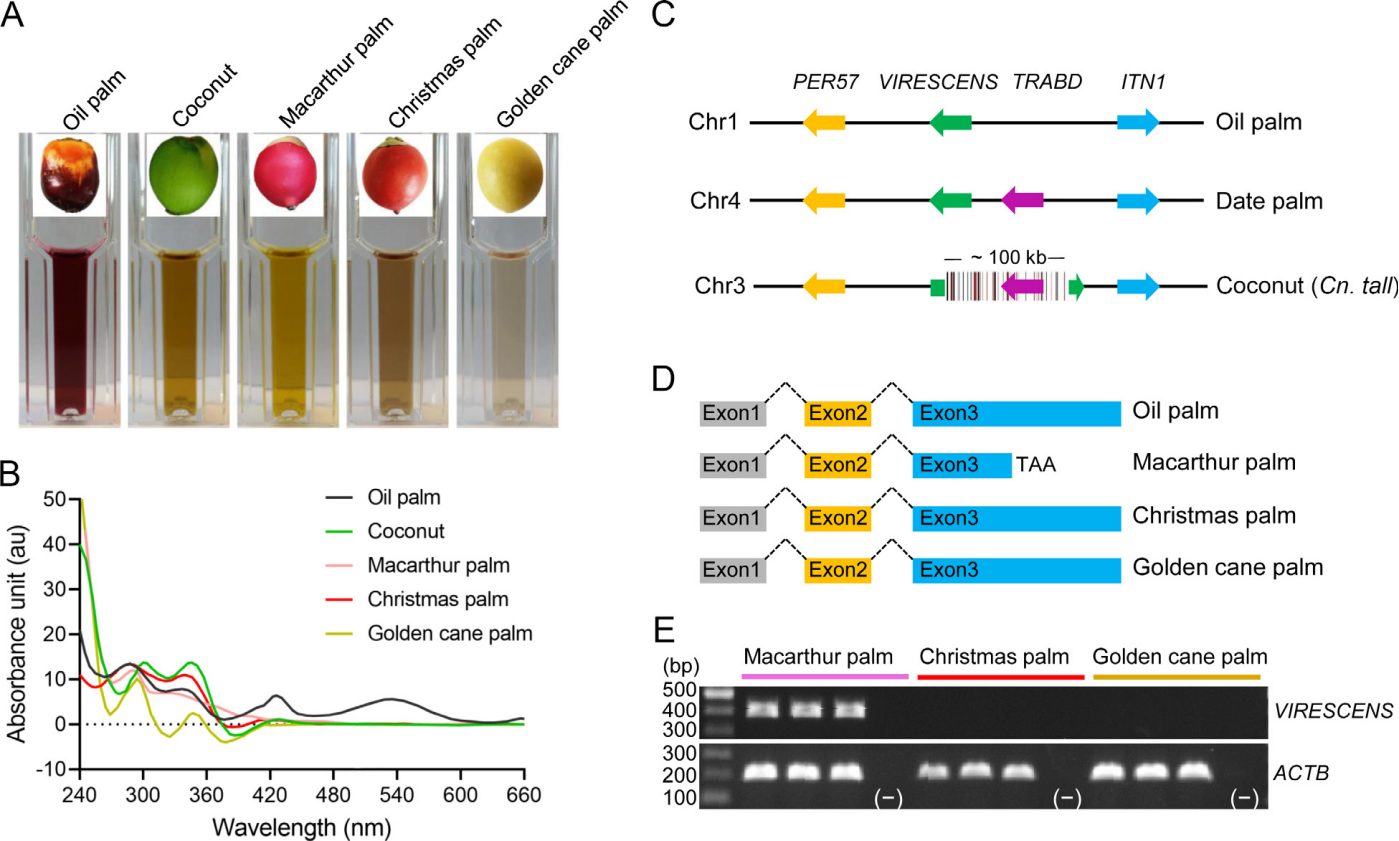
**Variations of the *VIRESCENS* gene in palms** **A.** Color of exocarp extracts in 1% acidified methanol across oil palm, coconut, Macarthur palm, Christmas palm, and golden cane palm. Oil palm *VIRESCENS* fruit type containing anthocyanins showed a dark purple color. **B.** UV absorption spectrum of exocarp extracts in 1% acidified methanol across palms. Oil palm *VIRESCENS* fruit type presented an absorbance peak at ∼ 530 nm, consistent with the absorption of anthocyanins. **C.** Genomic synteny of the *VIRESCENS* locus among oil palm, date palm, and coconut. Coconut *VIRESCENS* is disrupted by an insertion of a 100-kb highly repetitive sequence, where black and red bars indicate simple repeats and LTR retrotransposons, respectively. **D.** Gene models of the *VIRESCENS* gene in oil palm, Macarthur palm, Christmas palm, and golden cane palm. A premature termination codon was detected in exon 3 of Macarthur palm. **E.** Expression of *VIRESCENS* and *ACTB* (a housekeeping gene as a control) in Macarthur palm, Christmas palm, and golden cane palm, examined using gene-specific primers. Three individuals were examined for each species, and minus indicates negative control. UV, ultraviolet.

**Supplementary Figure S12:**
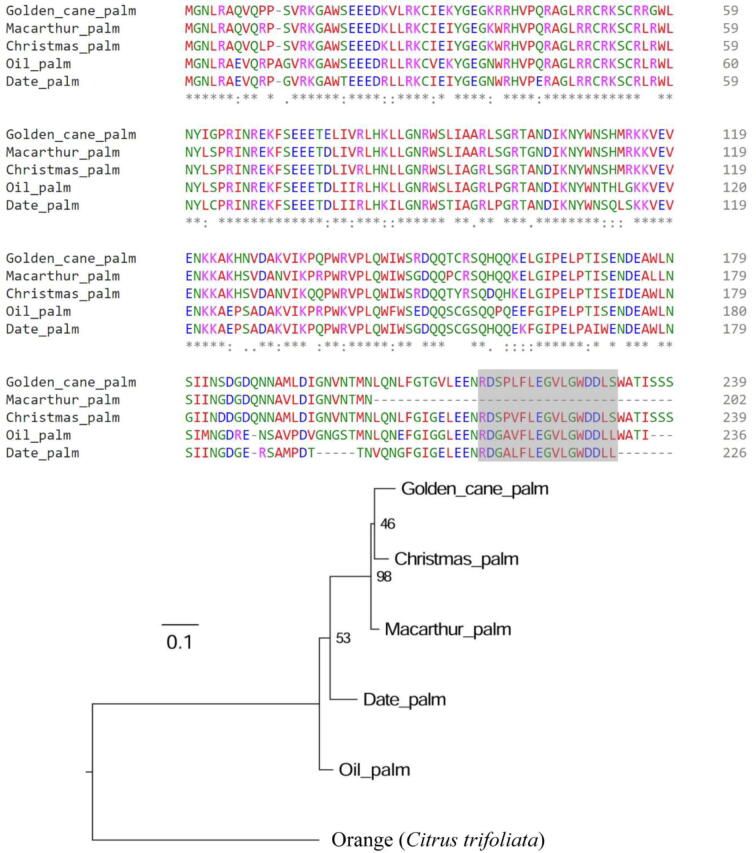
Alignment of VIRESCENS protein sequences and phylogenetic tree based on aligned protein sequences The studied species involved oil palm, date palm, Macarthur palm, Christmas palm, and golden cane palm. Predicted transcriptional activation domain of R2R3-MYB transcription factor was highlighted in grey shade. Amino acid mutation model of HIVb+I and 1000 bootstrap replications were used for tree construction. Orange (*Citrus trifoliata*) Myb transcription factor (ANI87841.1) was used as outgroup

It has been found that loss-of-function mutants of *VIRESCENS* in both oil palm and date palm is associated with loss of anthocyanins in exocarp, leading to conspicuousness of fruit colors [9], [21]. To examine the hypothesis, we cloned and analyzed the *VIRESCENS* gene of four additional palms: coconut, Christmas palm (*Adonidia merrillii*), Macarthur palm (*Ptychosperma macarthurii*), and golden cane palm (*Dypsis lutescens*). First, we measured the absorption spectrum of exocarp extracts and found that all four palms were deficient in anthocyanin accumulation in ripe fruit exocarp in contrast to the oil palm *VIRESCENS* fruit (wild-type) which had a high concentration of anthocyanins (Figure 3A and B). All coconut assemblies, including Catigan Green Dwarf [22], *Cn. tall*, and *Cn. dwarf* [23], were observed to harbor the complete *VIRESCENS* locus (Figure 3C). Interestingly, sequence analysis showed that the *VIRESCENS* gene in these genome sequences was consistently disrupted by an insertion of a highly repetitive region including ∼ 60 simple repeats and ∼ 50 LTRs, leading to loss of partial exon 1 and the whole exon 2 (Figure 3C). Disruption of *VIRESCENS* in coconut genome likely explains its green exocarp even in the ripe fruit. In Macarthur palm, we found a premature termination codon in the exon 3 of *VIRESCENS*, resulting in a predicted truncation of 34 amino acids in the C-terminal relative to the sequence of wild-type oil palm (Figure 3D). As predicted in both oil palm and date palm, the truncated 34 amino acids are overlapping with the transcriptional activation domain of R2R3-MYB transcription factor (Figure S12), and loss of this domain leads to deficiency in the regulation of anthocyanin accumulation [21]. In contrast, we did not identify evidence of loss-of-function mutations in the coding sequences of *VIRESCENS* for both Christmas palm and golden cane palm (Figure S12). However, the expression of this gene was undetectable in the ripe fruit exocarp of both palms (Figure 3E), implying that sequence variations in the regulation regions may have silenced *VIRESCENS* in these lineages. Taken together, our data suggest that variations in the *VIRESCENS* gene might be related to the conspicuousness of fruit colors in palms. Therefore, the *VIRESCENS* gene might be under selection by frugivores.

### Duplication of *PR* genes and their crucial roles in response to ***Ganoderma boninense*** infection in oil palm

**Figure 4:**
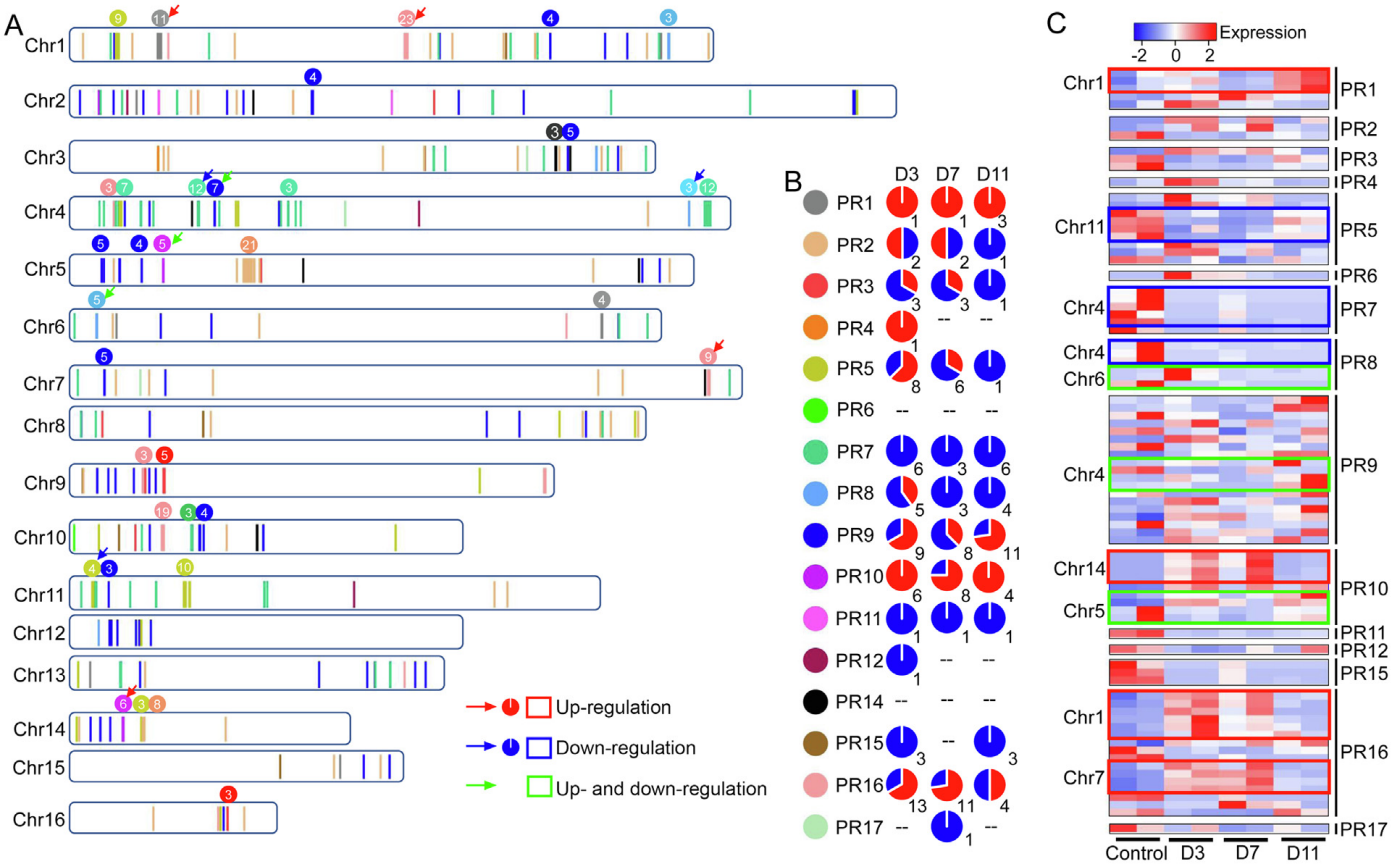
**Genome-wide distribution andrelative expression of*PR*genesin oil palm** **A.** Genome-wide distribution of *PR* genes throughout 16 chromosomes. Positions of *PR* genes are shown with vertical bars, and PR gene families are discriminated by colors as shown in (B). The number and family of *PR* genes in tandem arrays are indicated at the top of each array. Red and blue arrows indicate that *PR* genes in a certain tandem array show consistently up- and down-regulation, respectively, whereas green arrow indicates that *PR* genes in a certain tandem array show both up- and down-regulation, against fungal infection. **B.** Pie charts showing the numbers of differentially expressed *PR* genes (up-regulation in red, and down-regulation in blue) throughout 16 PR gene families in oil palm root at 3, 7, and 11 days post fungal infection. **C.** Heatmaps showing the relative expression of *PR* genes that are located in tandem arrays in oil palm root at 3, 7, and 11 days post fungal infection. Tandem arrays, within which *PR* genes show consistently up- and down-regulation, are highlighted with red and blue boxes, respectively, whereas those with *PR* genes showing both up- and down-regulation are highlighted with green boxes. PR, pathogenesis-related.

**Supplementary Figure S13:**
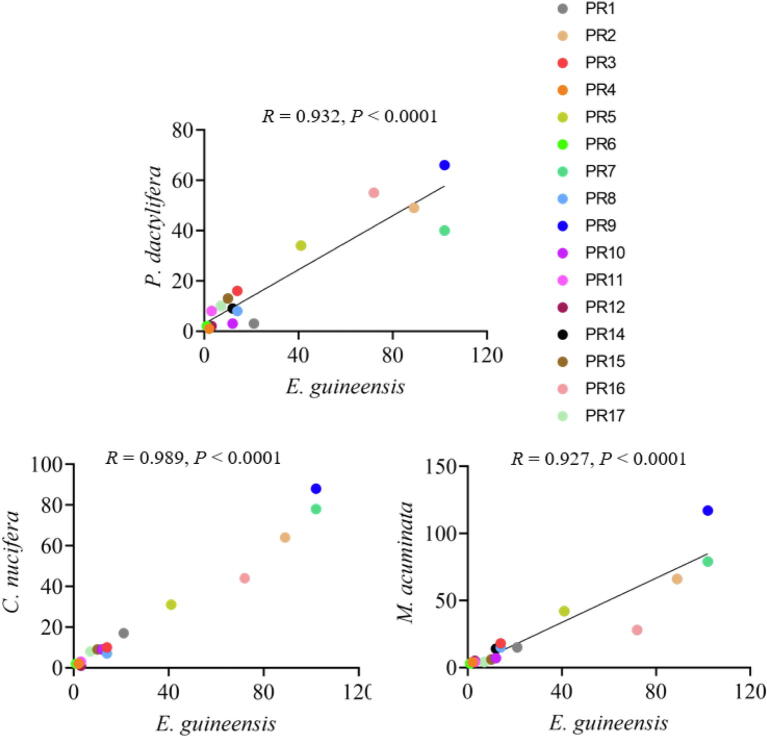
Correlation of the number of PR family members between species The correlation was estimated between the African oil palm and date palm, between the African oil palm and coconut, and between the African oil palm and banana

PR proteins, subgrouped into functionally different groups in plants, play critical roles in host defense to viral and fungal infections [24]. To date, little is known about the mechanism of these proteins responding to pathogen infections. We discovered 505 *PR* genes from 16 families in oil palm genome, among which 483 were mapped in 16 chromosomes (Figure 4A; Table S9). We found 319, 382, and 427 *PR* genes in date palm, coconut, and banana genome sequences, respectively. The size of gene families in oil palm was well correlated with that in date palm, coconut, and banana (*R* > 0.92, *P* < 1 × 10^−4^) (Figure S13), showing no significant evidence of expansion for a specific family. In oil palm, most of the *PR* genes presented in tandem duplications (Figure 4A). We defined a tandem array as a region within which genomic distance between any two adjacent *PR* genes was < 100 kb. We discovered 70 tandem arrays, with the number of *PR* genes in each ranging from 2 to 23. Over 64.4% (312) of *PR* genes were found to be located in tandem arrays. The largest tandem array was located at Chr1, consisting of 23 members of PR16 family (Figure 4A). We observed that ∼ 97% of *PR* genes in individual tandem arrays were resulted from tandem duplications, whereas the remaining ∼ 3% were from translocation or ancient duplication and divergence. Interestingly, we did not find obvious evidence that these tandem arrays were distributed between a pair of conserved syntenic chromosome blocks. These data suggest that *PR* genes are hyperactive in birth and death, as well as translocations, and may have frequently reorganized their genomic locations.

**Supplementary Figure S14:**
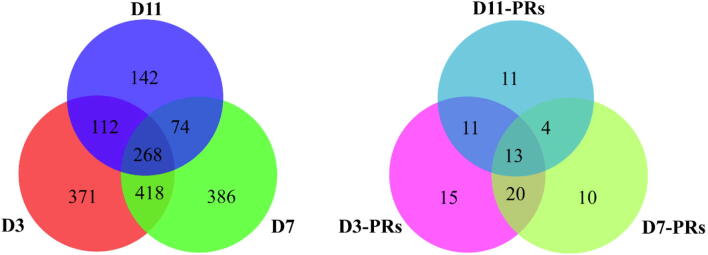
Identification of DEGs in the root samples DEGs were identified at 3, 7, and 11 days post infection with *Ganoderma boninense* (left) and the number of PRs that are identified as DEGs in these samples (right).

**Supplementary Figure S15:**
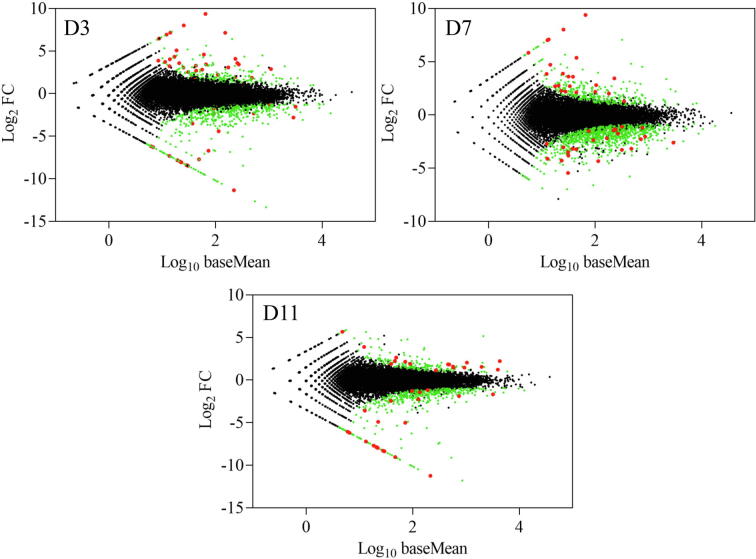
Total DEGs identified in the root samples DEGs were identified at 3, 7, and 11 days post infection with *Ganoderma boninense* (green dots) and PRs that are identified as DEGs in these samples (red dots).

**Supplementary Figure S16:**
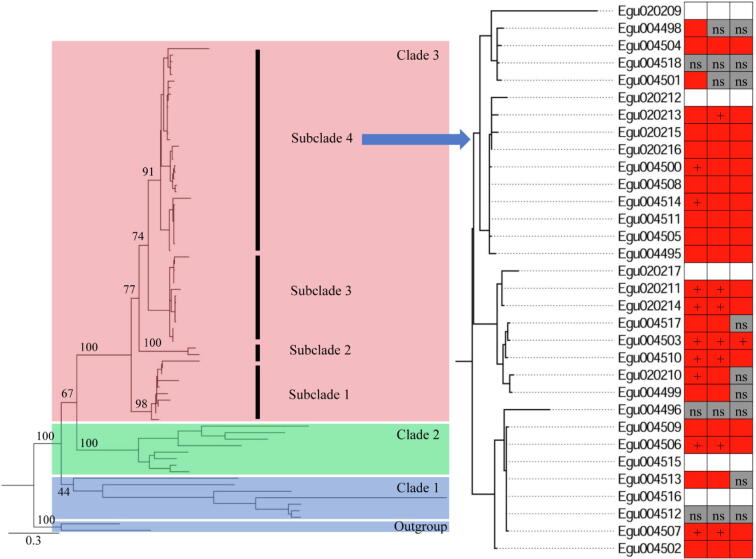
Phylogenetic tree of PR16 members and the relative expression of the PRs PRs that were classified into the subclade 4 were highlighted, where red shade indicates consistently up-regulated genes in all infected samples, regardless of statistical power, while plus indicates statistically significant DEGs. ns indicates non up-regulated genes

**Supplementary Figure S17:**
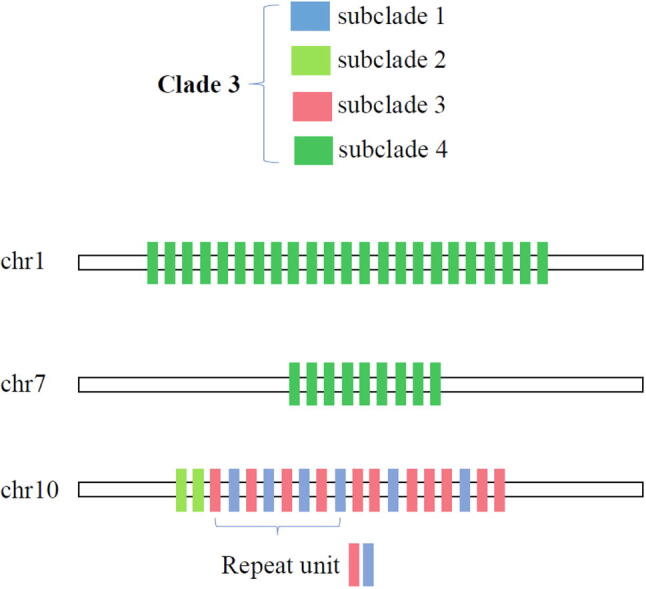
Genomic organization of PR16 members from different genetic clusters as revealed in Figure S18, in three tandem arrays at chr1, chr7, and chr10, respectively Different colors indicate different subclades

To understand more about the mechanism of pathogen defense in oil palm, we analyzed the genome-wide expression pattern of *PR* genes against the infection by *G*. *boninense* (Table S10) published by others [25]. We found that 84 (16.7%) *PR* genes were among the reported differentially expressed genes (DEGs) in root transcriptomes post infection (Figures S14 and S15; Table S11). The remaining *PR* genes may be induced in other tissues or involved in responses to the other pathogens. In detail, 59, 47, and 39 *PR* genes were detected as DEGs at 3, 7, and 11 days post infection (dpi), respectively (Figures S14 and S15). Thirty-eight (45.2%) DEGs were located in 10 tandem arrays, and distributed across seven chromosomes: Chr1, Chr4, Chr5, Chr6, Chr7, Chr11, and Chr14 (Figure 4A). DEGs in four (Chr1:PR1 members, Chr1:PR16 members, Chr7:PR16 members, and Chr14:PR10 members) and three (Chr4:PR7 members, Chr4:PR8 members, and Chr11:PR5 members) tandem arrays were consistently up- and down-regulated, respectively (Figure 4A). Analysis of DEGs in individual families (*e*.*g*., *PR* genes in PR5, PR9, and PR16 families) did not always show a consistent expression pattern (Figure 4B), suggesting neofunctionalization of the differentially expressed *PR* genes. Notably, DEGs belonging to the PR16 family were largely located in two tandem arrays at Chr1 and Chr7, in which all DEGs were up-regulated (Figure 4C), implying that these *PR* genes are subfunctionalized and involved in additive resistance to *G*. *boninense* [26]. Phylogenetic analysis revealed three major genetic clusters (Clades 1–3) in PR16 family, and the identified DEGs were all from the subclade 4, the youngest subclade of Clade 3 (Figure S16). Regarding another tandem array at Chr10, we found that PR16 members were from different subclades of Clade 3, and showed a chimeric pattern of organization. In this tandem array, *PR* genes of subclades 1 and 3 as a unit were repeatedly organized (Figure S17), as observed in some other plants, *i.e.*, *Theobroma cacao* and *Manihot esculenta* [27], [28], suggesting that *PR* genes have diverged prior to tandem duplications over evolutionary time. Taken together, our results reveal the crucial roles of large tandem arrays of *PR* genes in defense responses, particularly those consisting of evolutionary closely related *PR* genes. *PR* genes in chimeric tandem arrays or showing expression pattern shifts could have diverged over evolutionary time and likely been neofunctionalized and/or subfunctionalized.

### Population structure of the African oil palm

**Figure 5:**
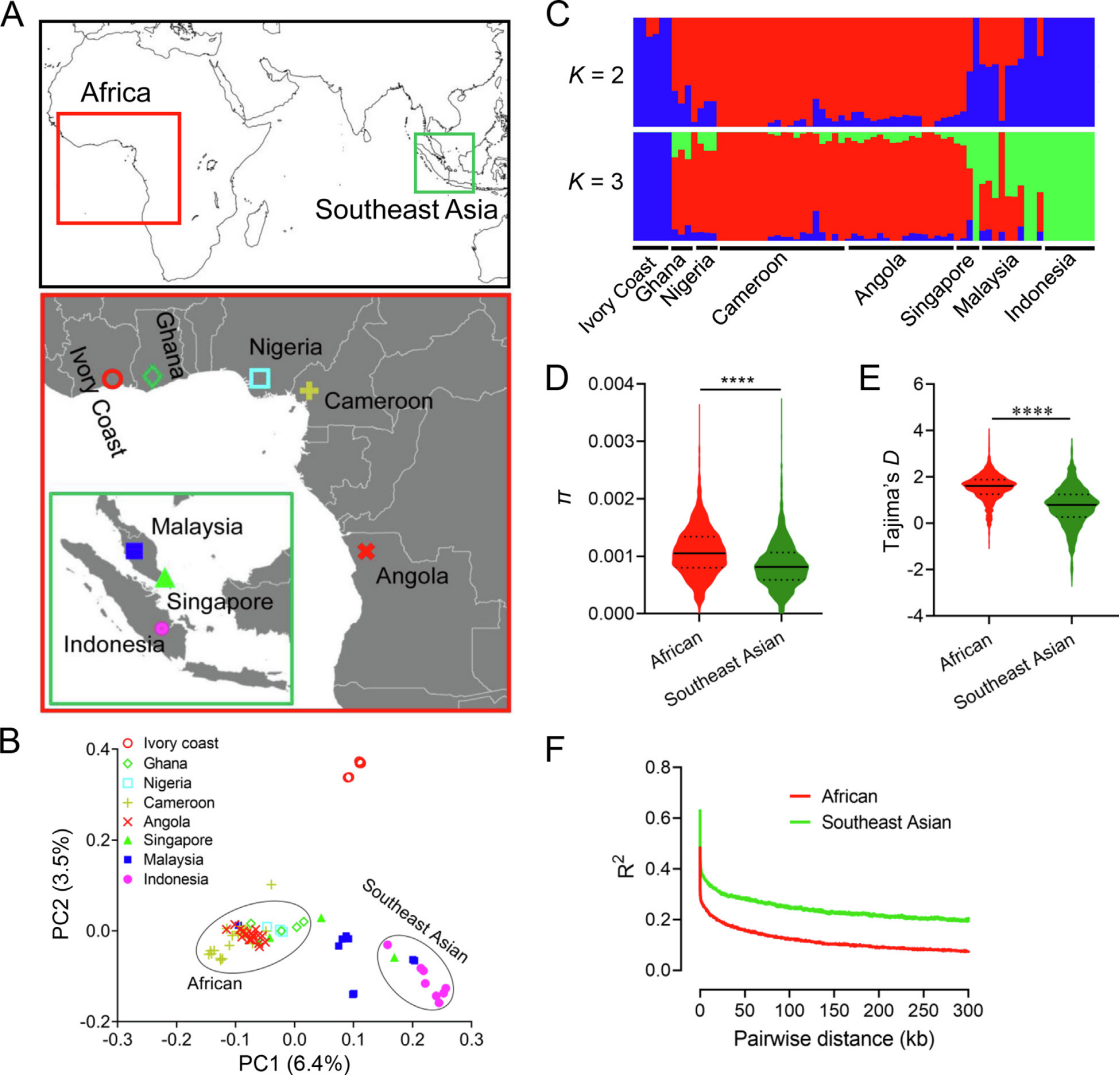
**Population divergence of oil palms** **A.** Sampling locations of the ancestral African oil palms and localized Southeast Asian oil palms. **B.** Population structure between and within African and Southeast Asian oil palms, revealed by PCA. **C.** Genetic clusters of population ancestry between and within African and Southeast Asian oil palms, inferred with admixture analysis. **D.** and **E.** Comparison of *π* and Tajima’s *D* between African and Southeast Asian oil palms, estimated with 100-kb window size. ****, *P* < 0.0001 (*t*-test). **F.** LD decay in African and Southeast Asian oil palms, respectively. PCA, principal component analysis; *π*, nucleotide diversity; LD, linkage disequilibrium; PC, principal component.

**Supplementary Figure S18:**
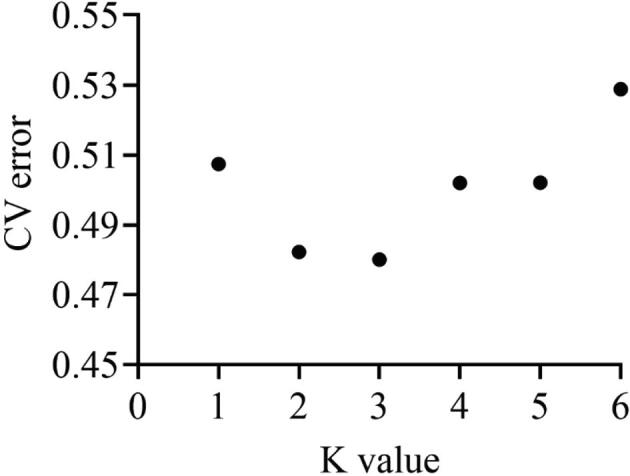
Inferring the most likely number of genetic clusters (K) among African and Southeast Asian oil palms, based on cross-validation error CV, cross-validation

We first examined the population structure of oil palms based on 4,410,076 single nucleotide polymorphisms (SNPs) generated by whole-genome resequencing of 72 trees (Figure 5A; Table S12). Both principal component analysis (PCA) and admixture analysis showed that the oil palms in Southeast Asia have clearly differentiated from their ancestral African ones except for those from Singapore and Malaysia, where most of the oil palms were either assigned into the African cluster or differentiated into an intermediate cluster between the African and Southeast Asian clusters (Figure 5B and C), since the first introduction in the 1840s [4]. Within Africa, oil palms from the Ivory Coast are strikingly differentiated from the remaining trees. Oil palms from Ghana, Nigeria, Cameroon, and Angola formed into the other cluster, in which pairwise differentiation among locations is limited, with an overall pairwise differentiation (*F*_ST_) of ∼ 0.05 (Table S13). The localized oil palms of Southeast Asia showed considerable differentiation among each other. Admixture analysis suggested the most likely number of genetic clusters to be three, followed by two (Figure S18). In agreement with PCA, admixture analysis showed evidence of a mixture of genetic clusters between the oil palms of Southeast Asia and Africa. The mixture occurred only in the oil palms of Singapore and Malaysia from Southeast Asia, implying repeated introduction of oil palms to Southeast Asia, likely as a result of escape of commercial cultivation or frequent commercial trade in the studied area.

Introduction of species would lead to loss of genetic diversity, as a result of founder effects and local selection in the new habitats. We examined the genetic diversity between African and Southeast Asian oil palms. Compared with their ancestral populations, oil palms in Southeast Asia showed significantly reduced genetic diversity, measured in nucleotide diversity (*π*: 0.0008 *vs.* 0.0011, *P* < 1 × 10^−48^, *t*-test) (Figure 5D), suggesting a recent bottleneck and/or local selection during establishment of the Southeast Asian populations. We also observed significantly more negative Tajima’s *D* in the Southeast Asian oil palms, in comparison to the African oil palms (*P* < 1 × 10^−72^, *t*-test) (Figure 5E), suggesting elevated positive selection in the localized Southeast Asian oil palms. We further estimated the linkage disequilibrium (LD) and found that LD decayed to half of the maximum within 10 kb in the oil palms of Africa, faster than that in Southeast Asia with a value of 30 kb (Figure 5F). This scale of LD allows for effectively identifying signatures of selection using genome-wide SNPs.

### Adaptive evolution of the African oil palm

**Figure 6:**
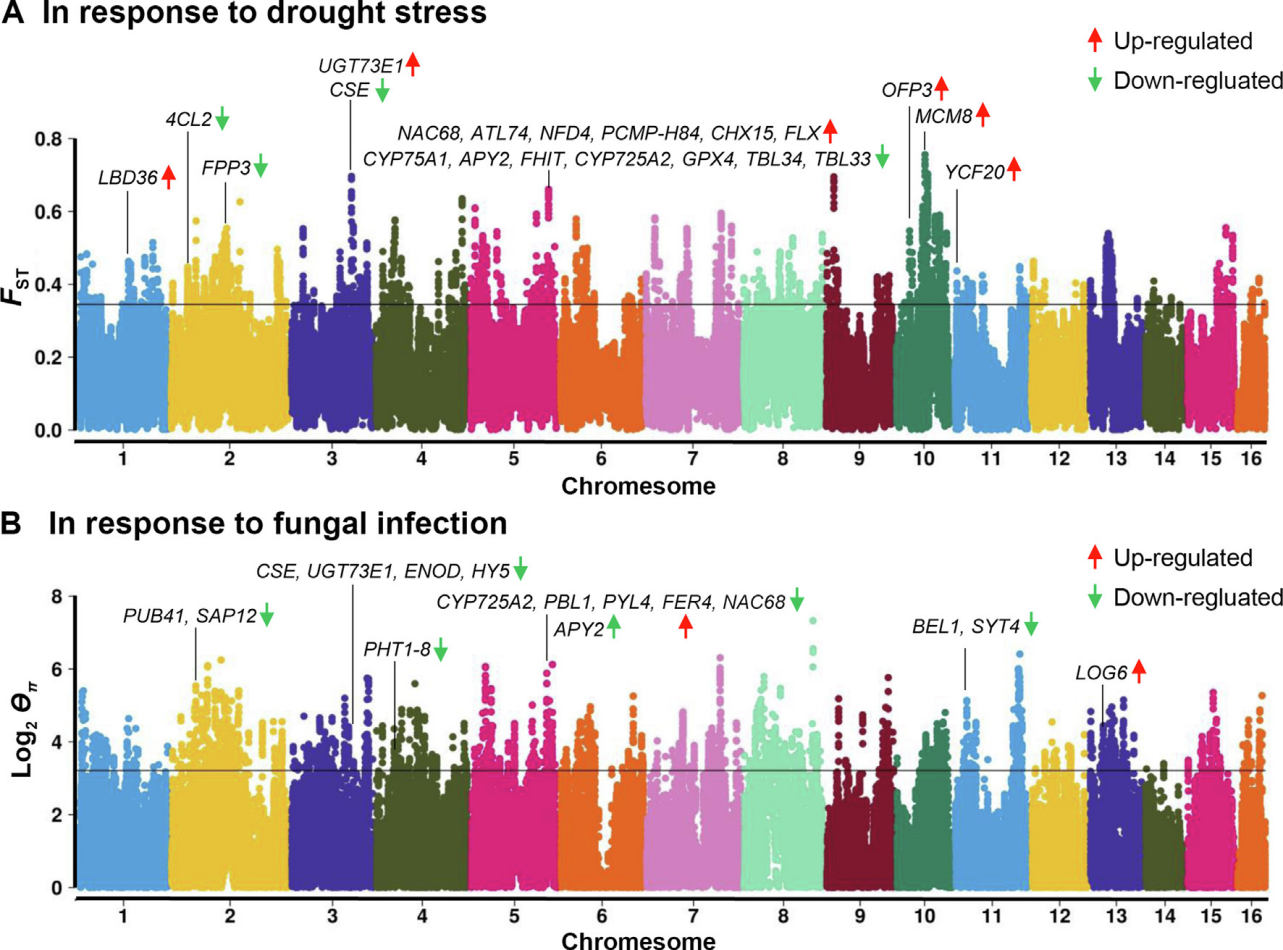
**Signaturesof selection in oil palm** **A.** Manhattan plot of genomic regions under putative selection, revealed by *F*_ST_ scanning between African and Southeast Asian oil palms. Genome-wide significance threshold at top 5% of windows in the empirical distribution is shown. Genes within outlier regions and identified as DEGs in root transcriptomes against drought stress are highlighted with gene names. Two *NFD4* genes are locally duplicated, and only one is indicated. **B.** Manhattan plot of genomic regions under putative selection, revealed by *ϴ_π_* scanning between African and Southeast Asian oil palms. Genes within outlier regions and identified as DEGs in root transcriptomes against fungal infection are highlighted. Two *APY2* genes are locally duplicated, and only one is indicated. DEG, differentially expressed gene; *F*_ST_, pairwise differentiation; *ϴ_π_*, the ratio of *π* values between ancestral and introduced populations.

**Supplementary Figure S19:**
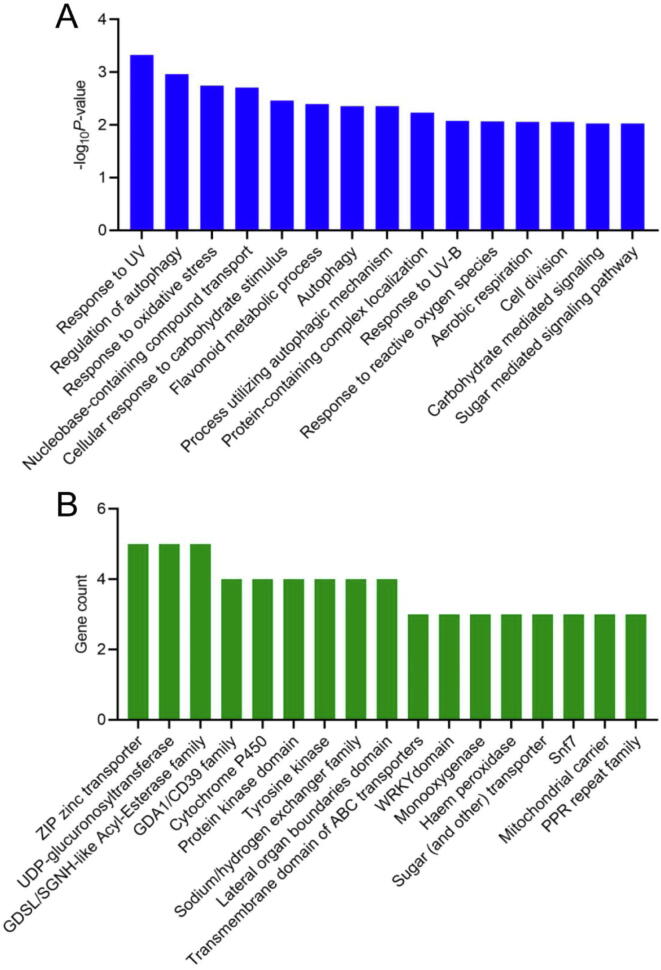
Gene Ontology (A) and Pfam enrichments (B) of the genes under putative selection, consistently identified by *F_ST_*, *ϴ_π_*, and Tajima’s *D* scans, respectively

To identify signatures of selection during introduction, we conducted a whole-genome scan for candidate regions between African and Southeast Asian oil palms. Both PCA and admixture analysis showed that the 72 oil palms were split into two major genetic clusters (Figure 5B and C). *F*_ST_ and the ratio of *π* values between ancestral and introduced populations (*ϴ_π_*) scans identified 127 consistent genomic regions under putative selection, with a total length of ∼ 23 Mb (1.3%) and containing 488 predicted protein-coding genes (Figure 6A and B). Sixty-four out of the 127 regions deviated from neutrality by Tajima’s *D* analysis. A total of 317 genes were identified in those regions. Only the consistent results of these genomic scans were considered for further analysis to obtain a confident and reliable result (Table S14). Gene Ontology enrichment analysis showed that these genes were more involved in stress responses, such as response to ultraviolet (UV), regulation of autophagy, and response to oxidative stress (Figure S19A). Enrichment analysis against the protein family database Pfam revealed that a notably large proportion of protein families were related to stress responses and disease defense, such as the GDA1/CD39 family, cytochrome P450, monooxygenase, and heme peroxidase. In addition, proteins belonging to the protein families of ZIP Zinc transporter, sodium/hydrogen, and transmembrane domain of ABC transporters that are related to ion transport were also enriched (Figure S19B). Three genes (two homologs of *WRKY70* and *WRKY24*) from the WRKY transcription factor family were under putative selection. Genes of this family have been extensively shown to be related to abiotic stress in both model plants and oil palm [29]. These results suggest that genomic regions under putative selection play an important role in the adaptive evolution of oil palm.

As the genes under putative selection are more involved in stress responses, in particular to pathogen infection and ion homeostasis, we separately analyzed these genes in correlation with DEGs responsible for resistance to *G*. *boninense* infection as described above and drought stress in our previous study [30]. Out of the 317 genes, 22 known protein-coding genes were revealed to be DEGs for drought stress, in which 12 and 10 genes were up- and down-regulated, respectively (Figure 6A; Table S15). Interestingly, we identified a selected region located at Chr5:105910001–110488835 bp with a length of ∼ 4.5 Mb, in which 13 (14.3%) out of 91 genes were DEGs, significantly higher than the ratio under null hypothesis of 4.3% throughout the whole genome. Most of these genes have been verified to be responsible for drought resistance in model plants, such as *4CL2*, *CYP75A1*, *APY2*, *NAC68*, *CHX15*, *TBL33*, and *TBL34* [31], [32], [33], [34]. Some of these genes under putative selection were also revealed to be responsible for heat stress, like *LBD36*, *NAC68*, *YCF20*, *4CL2*, and *CYP75A1* [35], [36], [37].

Among the putatively selected genes, 17 known protein-coding genes were identified as DEGs against *G*. *boninense* infection, in which 3 and 14 were up- and down-regulated, respectively (Figure 6B; Table S15). Some of these genes have been shown to associate with disease resistance in model plant species, such as *BEL1*, *PUB41*, *SAP12*, *CSE*, *NAC6*8, *PBL1*, and *PYL4* [32], [38], [39], [40], [41]. Interestingly, most of these genes were down-regulated against infection, implying a potential for decreased disease resistance in the oil palms of Southeast Asia; therefore, up-regulation of these genes may enhance disease resistance [41], [42]. Three genes, *CSE*, *NAC68*, and *CYP75A1*, were observed to be responsible for both drought tolerance and fungal resistance, suggesting that these genes play common roles in stress responses. Further functional studies of these genes could provide more useful insights into the adaptive evolution of the African oil palm and supply valuable resources for selective breeding of the species.

## Conclusion

We sequenced and assembled a chromosome-level genome of the African oil palm. The genome assembly is of high completeness and continuity, which will serve as a good reference genome for oil palm. Comparative genomic analysis reveals that historical transposon expansion, but not WGD, explains genome size variation of palms, providing essential resources for adaptive radiation. Sequence analysis of the *VIRESCENS* gene in palms suggests that DNA variations in this gene may be related to fruit colors. Moreover, highly tandemly repeated *PR* genes play an important role in defense responses to *Ganoderma* infection. Analysis of genetic variation between the ancestral African and recently introduced Southeast Asian oil palms identified signatures of selection, particularly on the introduced oil palms. Genes under putative selection are remarkably associated with stress responses, providing insights into adaptation to new habitats. The novel genomic resources and insights gained from this study could be exploited for comparative genomics, evolutionary studies, and genetic improvement of palms.

## Materials and methods

### Genome sequencing and assembly in oil palm

The same *Dura* tree, previously sequenced with Illumina platform [5], was sequenced using Single-Molecule Real-Time (SMRT) technology to improve the genome assembly. Genomic DNA was isolated using MagAttract HMW DNA Kit (Catalog No. 67563, Qiagen, Düsseldorf, Germany). Two 20-kb libraries were constructed and sequenced for > 150× coverage on PacBio Sequel II Sequencer (Pacific Biosciences, Menlo Park, CA) by BGI (Hong Kong, China). Flye v2.8 [43] was used to assemble the genome (-g 1.8 g -m 10,000 --asm-coverage 50 -i 3). Cleaned paired-end reads of 300-bp insert libraries and ∼ 100× coverage from Illumina sequencing [5] were used to polish the genome with Pilon [44].

### Construction of high-density linkage maps

For construction of high-density linkage maps, five F_2_ families consisting of a total of 978 progenies were used for RAD sequencing. DNA was isolated from leaves of each tree using DNeasy Plant Mini Kit (Catalog No. 69104, Qiagen). DNA was digested with *Pst*I-HF restriction enzymes (Catalog No. R3140L, New England Biolabs, Ipswich, MA), and RAD libraries were constructed as described in our previous study [45]. The libraries were sequenced for 150-bp single-end reads on NextSeq500 platform (Illumina, San Diego, CA). Raw reads were cleaned with process_radtags (-r -c -q -t 130) in Stacks package [46]. Cleaned reads of ∼ 7.3 million for each sample were aligned to the aforementioned reference genome with BWA-MEM [47] with default parameters. Aligned reads were assembled and called for SNPs with Stacks package [46], according to our previous study [45]. Only one SNP from each RAD tag was kept. SNPs that were present in > 90% individuals within each family and showed Mendelian segregation distortion of > 0.05 in Chi-squared test were retained. Linkage mapping was carried out using Lep-MAP3 [48], with a logarithm of the odds score (LOD) of > 10 for linkage group assignment.

### Construction of chromosomal-level genome assemblies of palms

RAD tags that were incorporated into the five high-density linkage maps were aligned to the contigs to assign genomic coordinates. Chimeric contigs were determined by linkage maps, which are not likely to have among-chromosome grouping errors [49]. Contigs with more than four markers mapped to different linkage groups, were considered as chimeric and were then split at the longest gaps between mismatched fragments. The program ALLMAPS [50] was then employed to anchor contigs to linkage maps, with default parameters. Centromere positions were estimated based on the distribution of recombination rates along individual chromosomes. Recombination rates, measured as ρ = 4Ner per kb, were estimated using LDhat [51]. Completeness of genome was examined by mapping to BUSCO v3.0.1 database [52].

### Repeat and genome annotations

RepeatModeler (https://www.repeatmasker.org) was first used to build a custom repeat library of the studied species. RepeatMasker [53] was then employed to identify repetitive sequences based on the custom repeat library and Repbase database [54]. Tandem repeats were further annotated using Tandem Repeats Finder [55]. Finally, we combined and filtered these repetitive sequences to obtain the nonredundant repeat annotations of the genome based on the coordinates. Assessment of the intact LTR retrotransposons was carried out using LTR_retriever [56]. Demographic history of the TEs was inferred by investigation on the most abundant LTRs. One hundred LTRs were randomly selected from 40 random subfamilies of *Copia*. Full sequences were extracted and aligned with MUSCLE [57]. The distribution of pairwise sequence similarity within a family was used to estimate the temporal dynamics of TE activity.

Genome was annotated with MAKER2 pipeline [58]. Genome sequences were first soft-masked using RepeatMasker [53], based on the aforementioned repetitive libraries. Cleaned mRNA sequencing reads of multiple organs from our previous study [5] were assembled with Trinity [59] and used for evidence-based annotation. For *ab initio* gene model prediction, protein sequences of *E. guineensis* EG5.1 [6] and EGv2 [5], date palm Barhee BC4 [9], and coconut HainanTall [60] were used as evidence. SNAP [61] and AUGUSTUS [62] were iteratively used to train gene models. Predicated gene models that contained TE domains and were not supported by transcripts were filtered. Cleaned gene models were then annotated by BLAST to Non-Redundant Protein Sequence Database and RefSeq databases with BLASTP (E-value < 1E–10).

### Evolutionary analysis

Homologous blocks within and between species of interest were determined by pairwise whole-genome alignment with LASTZ [63] and all-versus-all BLASTP search with Ortholog-Finder at gene level [64]. Putative one-to-one orthologs and paralogs from a pair of homologous blocks between oil palm and date palm and within oil palm, respectively, were aligned using MUSCLE [57]. Coding sequences were then aligned with the guidance of corresponding protein alignments. DNA alignments were further polished using trimAl [65]. *Ks* was estimated between a pair of homologous genes using KaKs_Calculator [66]. Local duplicated genes were identified by analyzing the results of all-versus-all BLASTP search as described above, based on their genomic coordinates. To estimate the sequence divergence of LTR retrotransposons, 100 members from each of the randomly selected 30 *Copia* subfamilies were randomly selected and pairwise aligned, according to a previous study [15]. Pairwise sequence divergence was estimated and compared with that of homologous genes to infer the relative evolution time [15].

### Transcriptome analysis

To compare the expression patterns of homologous genes, RNA-seq reads of various parts from oil palm in our previous studies [30], [67], [68] and from date palm [69] were reanalyzed. Raw reads were cleaned with process_shortreads in Stacks package with default parameters, to remove adaptors and low-quality reads. Cleaned reads were then aligned, with default parameters, to the reference genome using STAR [70]. Uniquely mapped reads were counted to calculate gene expression level based on genome annotations, using the program HTSeq-count [71]. Gene expression level was then quantified as the number of fragments per kilobase of transcript per million mapped reads (FPKM). Heatmapper [72] was used to visualize the clusters and relative expression of genes.

### Analysis of ***VIRESCENS*** in palms

The presence of anthocyanins across palms was examined by measurement of the absorption spectrum of exocarp extracts in 1% acidified methanol, according to a previous method [21]. Equal exocarp material (100 mg) for each palm were used for extraction and the spectrum of UV absorption was measured from 240 nm to 700 nm with a 10-nm interval. Sequences of *VIRESCENS* across the studied palms were amplified either by amplification of genomic DNA or complementary DNA (cDNA), using primers designed according to sequence homology among oil palm, date palm, and coconut and primer walking (Table S16). Coding sequences were predicted based on oil palm *VIRESCENS* gene [21]. Predicted protein sequences were aligned using MUSCLE [57], and a phylogenetic tree was constructed using IQ-TREE2 [73], under the model of HIV between-patient matrix HIV-Bm with a proportion of invariable sites (HIVb+I) with 1000 bootstrap replications. The relative expression of *VIRESCENS* was examined using reverse transcription polymerase chain reaction (RT-PCR). In brief, total RNA extraction and cDNA synthesis were carried out according to our previous study [30]. cDNA corresponding to 50 ng of total RNA was used as template for amplification using gene-specific primers, and the housekeeping gene, *ACTB*, was used as a reference, with the following PCR condition: 94 °C for 5 min, followed by 35 cycles of 94 °C for 30 s, 60 °C for 30 s, and 72 °C for 30 s, and a final extension of 72 °C for 5 min. RT-PCR products were examined by running 2% agarose gel.

### Characterization of PR proteins

Protein sequences of all PR family members of different plant species [27] were used as baits to search the genomes of oil palm, date palm, coconut, and banana, with BLASTP (E-value < 1E–5). Protein sequences were extracted and manually curated, and were then sorted and classified based on protein domains, according to a previous study [27]. Genomic coordinates of *PR* genes in oil palm were extracted from annotation files to study the distribution and duplication patterns. Protein sequences of PR family members of interest were aligned using MUSCLE [57]. Alignments were refined using trimAl [65]. Phylogenetic trees were constructed using IQ-TREE2 [73], under automatically searched mutation model (JTT+R4).

Functions of PR proteins in disease resistance were studied by analyzing the RNA-seq data set of oil palm seedlings infected with *G*. *boninense* inoculums at 3, 7, and 11 dpi [25]. Processing of raw sequencing reads, alignment to reference genome, and count of mapped reads were carried out as described above. Normalization of transcripts and identification of DEGs were performed using DESeq2 [74]. Genes with a fold change > 2 and a significant cutoff value of 0.005, corresponding to 0.1 after false discovery rate (FDR) corrections, were considered as DEGs.

### Whole-genome resequencing and variant calling

A total of 72 trees from West Africa (50) and Southeast Asia (22) were selected for sequencing. Libraries of 550-bp inserts were constructed using Truseq DNA PCR-Free Kit (Catalog No. 20015963, Illumina) and sequenced on NextSeq500 (Illumina). Raw reads were filtered as described above. Cleaned reads were aligned against reference genome using BWA-MEM [47], and variants were called using the Picard/GATK v4.0 best practices workflows [75]. We further filtered SNPs with the parameters: “QD < 2.0 || DP > 5 || FS > 60.0 || MQ < 40.0 || MQRankSum < −12.5 || ReadPosRankSum < −8.0 || SOR > 4.0”. Only SNPs were retained for further analysis, and those with missing data across populations > 20 were also removed.

### Genetic diversity and population structure analyses

Population genetic diversity indexes including *π*, Tajima’s *D*, and *F*_ST_ were estimated using VCFtools [76]. Population structure was analyzed with PCA using PLINK2 [77]. Genetic clusters from ancestry were inferred using ADMIXTURE [78], with the number of clusters ranging from 2 to 10. Cross-validation error was estimated to determine the most likely number of ancestral populations. LD between SNPs within populations (R^2^) was estimated using PopLDdecay (-MAF 0.02, -Het 0.88, -Miss 0.25) [79].

### Identification of signatures of selection

Signatures of selection between populations were inferred using *F*_ST_, *ϴ*_*π*_, and Tajima’s *D* statistics within the Southeast Asian samples. These estimates were calculated in sliding window size of 100 kb with a window size step of 50 kb. Genomic regions consistently within top 5% of windows for *F*_ST_ and π, and bottom 5% of windows for Tajima’s *D* in the empirical distribution, were considered as outliers under putative selection. Protein-coding genes in outlier regions were considered under putative selection. Protein sequences were extracted and annotated against *Arabidopsis* protein database (Ensembl TAIR10) using BLASTP with an E-value cutoff < 1E–10. Metascape [80] was employed to perform Gene Ontology enrichment analysis, with *Arabidopsis* as reference, using default parameters. DEGs responding to drought stress [30] and fungal infection as described above were used to infer signatures of selection under putative stresses.

## Data availability

Raw sequencing reads generated in this study have been deposited in the DNA Data Bank of Japan SRA database (BioProject ID: PRJDB11628) which are publicly accessible at https://www.ddbj.nig.ac.jp, and also have been deposited in the Genome Sequence Archive [81] at the National Genomics Data Center (NGDC), Beijing Institute of Genomics (BIG), Chinese Academy of Sciences (CAS) / China National Center for Bioinformation (CNCB) (GSA: CRA008676) which are publicly accessible at https://ngdc.cncb.ac.cn/gsa. The chromosomal-level genome sequences of oil palm can be accessible at https://genhua.tll.org.sg/, and also have been deposited in the China National GeneBank DataBase (CNGBdb: CNA0047477) which are publicly accessible at https://db.cngb.org, and the Genome Warehouse [82] at the NGDC, BIG, CAS / CNCB (GWH: GWHBKAS00000000) which are publicly accessible at https://ngdc.cncb.ac.cn/gwh.

## Competing interests

Yuzer Alfiko, Rahmadsyah Rahmadsyah, Sigit Purwantomo, and Antonius Suwanto are current employees of Wilmar International Ltd. All the other authors have declared no competing interests.

## CRediT authorship contribution statement

**Le Wang:** Methodology, Software, Formal analysis, Visualization, Writing – original draft, Writing – review & editing. **May Lee:** Resources, Methodology, Formal analysis. **Zi Yi Wan:** Resources, Methodology, Formal analysis. **Bin Bai:** Resources, Methodology, Formal analysis. **Baoqing Ye:** Software, Formal analysis. **Yuzer Alfiko:** Resources, Methodology. **Rahmadsyah Rahmadsyah:** Resources, Methodology. **Sigit Purwantomo:** Resources, Methodology. **Zhuojun Song:** Resources, Formal analysis. **Antonius Suwanto:** Resources, Supervision. **Gen Hua Yue:** Conceptualization, Supervision, Resources, Funding acquisition, Writing – review & editing. All authors have read and approved the final manuscript.

## Supplementary material

Supplementary Table S1 Summary statistics of genome assemblies of oil palm, date palm, and coconut

Supplementary Table S2 Summary statistics of five high-density linkage maps, including the number of SNPs and length of each linkage group

Supplementary Table S3 Summary statistics of SNPs that are mapped to contigs and genomic sequences that are anchored onto the linkage maps and oriented

Supplementary Table S4 Genomic sequences anchored onto 16 linkage groups/pseudochromosomes of the African oil palm genome

Supplementary Table S5 Completeness of oil genome assemblies assessed by BUSCO

Supplementary Table S6 Summary statistics of transposable elements in the African oil palm genome

Supplementary Table S7 Summary statistics of LTR retrotransposon superfamilies in the African oil palm genome and date palm genome

Supplementary Table S8 RNA sequencing samples used to examine homologous gene expressions in African oil palm genome

Supplementary Table S9 Summary statistics of PRs identified in the genome of oil palm, date palm, coconut, and banana

Supplementary Table S10 Root samples of African oil palm at 3, 7, and 11 days post infection with *Ganoderma boninense* and negative controls

Supplementary Table S11 Differentially expressed PRs from different families at 3, 7, and 11 days post infection with *Ganoderma boninense*

Supplementary Table S12 African oil palm trees used for whole-genome resequencing and the sequencing coverage, sample location, and date for each tree

Supplementary Table S13 Pairwise *F_ST_* of seven African oil palm samples, based on whole genome resequencing

Supplementary Table S14 Genes identified under putative selection in the oil palm genome

Supplementary Table S15 Genes that were identified under putative selection in the genome and to be differentially expressed in transcriptomes of oil palm against drought stress or fungal infection

Supplementary Table S16 Primers used to amplify and examine the expression of VIRESCENS gene in palms
